# SpatialRNA: a Python package for easy application of Graph Neural Network models on single-molecule spatial transcriptomics dataset

**DOI:** 10.1093/bioinformatics/btaf659

**Published:** 2025-12-13

**Authors:** Ruqian Lyu, Annika Vannan, Jonathan A Kropski, Nicholas E Banovich, Davis J McCarthy

**Affiliations:** Bioinformatics and Cellular Genomics, St Vincent’s Institute of Medical Research, 9 Princes Street, Fitzroy, Victoria, 3065, Australia; Colonial Foundation Diagnostics Centre, The Walter and Eliza Hall Institute of Medical Research, VIC 3052, Parkville, 3050, Australia; Translational Genomics Research Institute, Phoenix, AZ 85004, United States; Division of Allergy, Pulmonary and Critical Care Medicine, Department of Medicine, Vanderbilt University Medical Center, Nashville, TN, 37232, United States; Department of Cell and Developmental Biology, Vanderbilt University, Nashville, TN 85004, United States; Department of Veterans Affairs Medical Center, Nashville, TN, 37212, United States; Translational Genomics Research Institute, Phoenix, AZ 85004, United States; Bioinformatics and Cellular Genomics, St Vincent’s Institute of Medical Research, 9 Princes Street, Fitzroy, Victoria, 3065, Australia; Faculty of Medicine, Dentistry and Health Sciences, University of Melbourne, Parkville, VIC, 3010, Australia

## Abstract

**Summary:**

Image-based spatial transcriptomics (iST) deliver gene expression measurements of RNA transcripts in tissue slices with single-molecule resolution and spatial context preserved. Modern Graph Neural Network (GNN) models are promising methods for capturing the complex molecular and cellular phenotypes in tissues at single-transcript and single-cell levels. A key application of GNNs is the detection of spatial domains or niches, that is, groups of molecules and/or cells that collaboratively work together to produce complex phenotypes. Due to the vast number of detected transcripts in (iST) dataset, applying GNNs on RNA molecule graphs is not trivial. We present a Python package, SpatialRNA, for easy (sub)graph generation from tissue samples and provide comprehensive tutorials for convenient and efficient application of Graph Neural Network models under the PyG framework. This highly scalable tool comprehensively segments tissue into spatial domains, aiding in biological interpretation of iST data and its underlying molecular microenvironments.

**Availability and implementation:**

The SpatialRNA package is freely accessible from online repository https://github.com/ruqianl/spatialrna and can be installed via pip. Comprehensive tutorials, guidance on parameter selection, and complete workflows of case studies are available from the documentation website https://ruqianl.github.io/spatialrna_docs/, and uploaded on Zenodo with a DOI 10.5281/zenodo.17339575.

## 1 Introduction

Image-based spatial transcriptomics (iST) platforms are able to detect RNA transcripts within the spatial context of tissues, allowing quantification of gene expression in cells and tissues in a spatially-aware manner. These platforms, such as MERFISH (Vizgen) (Chen *et al.* 2015), seqFISH (Lubeck *et al.* 2014, Eng *et al.* 2019), and Xenium (10x Genomics), commonly build upon Fluorescence in situ hybridization (FISH) techniques that visualize RNA locations through fluorescent-dye-attached probes designed to specifically bind to transcript targets. With iST platforms advancing, hundreds to thousands of gene species are measured in parallel in a given tissue sample, yielding high-throughput single-molecule resolution spatial gene expression data.

Broadly, there are two analysis approaches for iST data: cell-based and cell-free approaches (He *et al.* 2021, Si *et al.* 2024). With cell-based approaches, cell segmentation (Stringer *et al.* 2021, Wang *et al.* 2023, Fu *et al.* 2024) is required to obtain cell boundaries using auxiliary imaging data such as nuclei and cell membrane staining as well as gene expression profiles. Cell-level gene expression is then derived based on cell boundaries through summarizing molecules within cell boundaries, which allows application of many existing single-cell analysis methods. Performance of cell-based analysis is heavily influenced by the quality of cell segmentation, and errors in cell segmentation introduce biases in the final outcome. Cell-free analysis methods perform modelling directly on the detected transcripts and their spatial neighbourhoods for cell or tissue typing (He *et al.* 2021, Si *et al.* 2024). Cell-free methods are suitable for iST data without reliable cell segmentations, e.g. iST data without quality membrane staining images, or tissues with cells that are particularly hard to segment due to irregular shapes or high cell density. Cell-free methods can thus serve as a complementary approach to cell-based analyses when cell segmentation is imperfect.

Graph Neural Network (GNN) models are deep machine learning methods that are designed to work with graph structured data. These methods also show great potential in cell-free iST data analysis, with specific GNN models such as GraphSAGE (Hamilton *et al.* 2017) having been successfully applied to spatial RNA graphs for learning diverse spatial organization of RNA transcript neighbourhoods (i.e. spatial domains or ‘niches’) (Medaglia *et al.* 2017, Partel and Wählby 2021, Mason *et al.* 2024, Vannan *et al.* 2025). Characterizing spatial niches in the tissue is helpful for understanding mechanisms of how groups of molecules or cells work together to perform complex functions and is essential for associating disease phenotypes with coordinated changes in molecular and cellular compositions (Partel and Wählby 2021, Vannan *et al.* 2025). To apply GNNs on iST data in a cell-free (molecule-based) manner, spatial RNA graphs are constructed using RNA molecules as nodes, and edges are added to connect RNA molecules to their spatial neighbours. Input nodes in the graph are associated with an initial feature vector, usually a one-hot-encoding representation of the gene labels. GNNs are then deployed to learn functions of aggregating neighbourhoods of individual molecules to project each molecule onto a latent space where molecules with similar neighbourhood compositions are closely embedded in similar locations in the latent space. Based on the latent embeddings of molecules, we derived molecule-based clusters, which we refer to as molecular niches in this work, representing spatially coordinated microenvironments of molecules.

However, applying GNNs on RNA molecule graphs, especially in large tissue samples (both large in physical dimension of each tissue sample and/or large in sample size), is not trivial due to the vast number of RNA molecules detected, at the scale of millions of transcripts. We recently introduced our working GNN computational workflow on large-scale data, which used sampled subgraphs, in a pulmonary fibrosis study involving 45 lung tissue samples totalling 299 million RNA transcripts (Vannan *et al.* 2025). The workflow consisted of multiple steps with designated Python scripts for graph construction, subgraph sampling, and model training, and it was able to detect and comprehensively segment spatial tissue niches across healthy and fibrotic lungs in a cell-free manner, allowing for biological insights into how niches change across disease states and correspond to physician-annotated pathologies among other downstream analysis (Vannan *et al.* 2025). In this work, we adapted and improved the previous computational workflow to make the application of GNNs on such iST data easier and more streamlined. We hereby introduce SpatialRNA, a new Python package that integrates the multiple processing components [e.g. sample tiling, (sub)graph constructing, on-disk data loading, model training, inference, clustering and visualization functions] into a unified framework designed for the convenient application of existing and novel GNN architectures on iST samples using the modern PyG library (Fey and Lenssen 2019, Fey *et al.* 2025).Furthermore, the current framework can be adapted with new training objectives for solving other challenges in iST data analysis at transcript-level.

## 2 Methods and implementations

**Figure 1.**
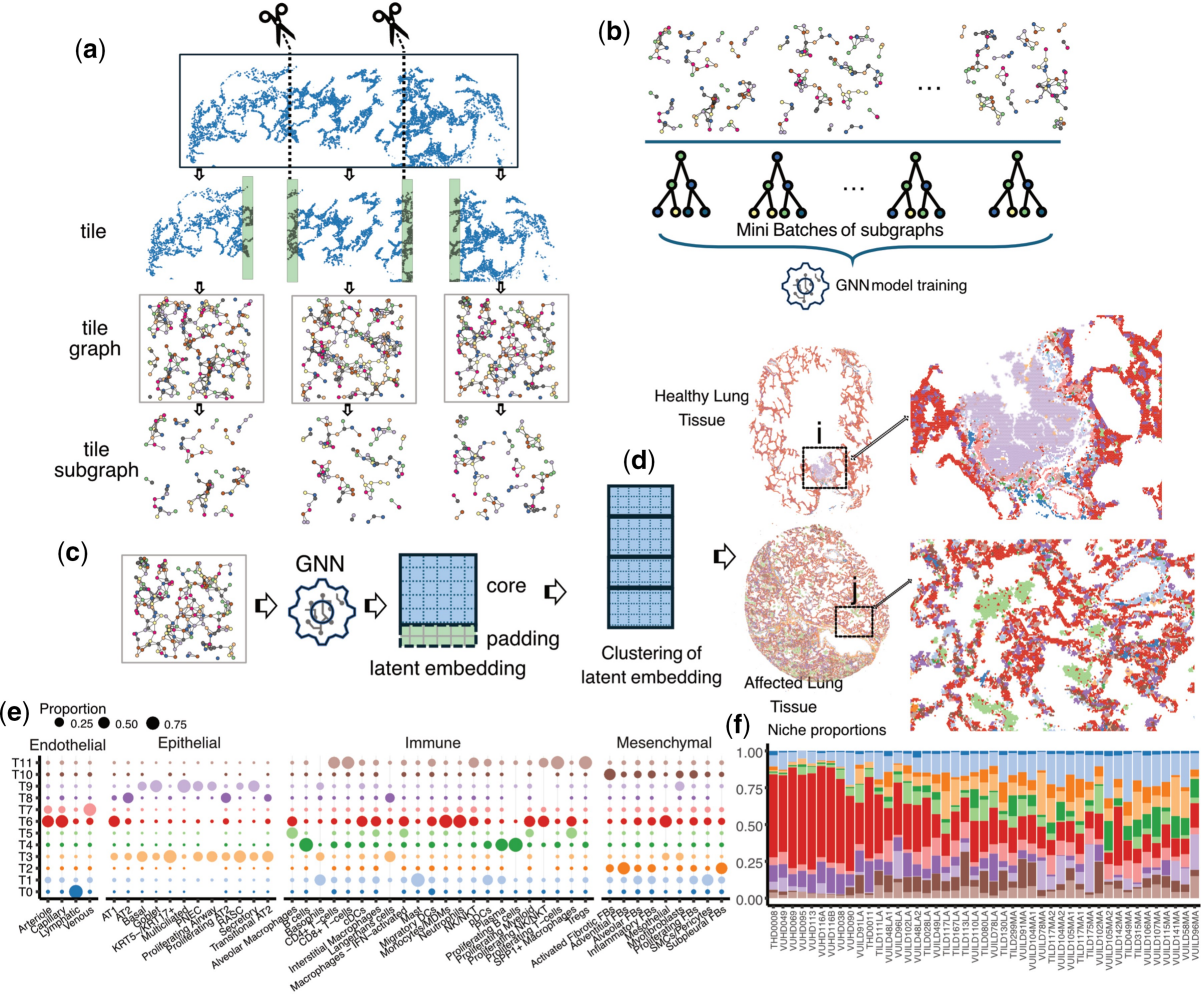
SpatialRNA for easily tiling large tissue samples, constructing tile (sub)graphs, and facilitating mini-batched GNN model training. (a) An example tissue is tiled into three tissue tiles. Each tile consists of a core tissue area as well as padding areas to include neighbours of transcripts located on the edges of the tiles. Subgraphs are sampled from the tile graph, and all subgraphs from multiple tissues (tissue tiles) were merged for model training as shown in b. A variation in the process is to load tile graph in small batch and omit the subgraph sampling and merging step (Case study 2). (b) Mini-batches of graphs were generated from the merged graph for GNN (e.g. GraphSAGE, GAT, GATv2) model training. (c) Nodes (i.e. transcripts) in each tissue tile graph were projected onto the latent space using the trained GNN model. Nodes in the padding areas are discarded. (d) Nodes from multiple tiles of tissues were stacked and clustering was applied to the embedding matrix for partitioning the transcripts into groups. Two lung tissues from Case Study 1, were visualized after hex bin aggregation, with highlighted areas in a healthy lung i (showing an airway structure captured by T9) and an affected lung tissue j (showing a macrophage accumulation in niche T5). (e) The cellular composition of the 12 identified molecular niches (T0–T11) from the idiopathic pulmonary fibrosis (lung) case study 1. (f) The composition of molecular niches across 45 tissues ordered by their percent of pathology with disease-emergent or enriched in disease niches identified, including immune (T4, T5, T11) and fibrotic (T10).

The goal of applying GNNs for cell-free molecule-based analysis for iST data for spatial niche identification is to learn new representations of individual molecules. Specifically, the GNN assigns a latent embedding vector for each individual molecule based on the composition of its spatial neighbourhood (defined by a distance threshold). The latent embeddings of molecules can be subsequently used for unsupervised clustering to detect spatial tissue domains across samples (Fig. 1). Since the aim is to identify spatial tissue niches in an unsupervised fashion, there are no classification labels available for the nodes in the graph. Therefore, GNNs cannot be trained in a node classification setup. Instead, the GNNs models are trained by solving a link (or edge) classification task. This task involves predicting whether a link exists between a pair of nodes based on the similarity of the profiles of their spatial neighbours, which is supervised by the graph structure (Hamilton *et al.* 2017, Partel and Wählby 2021, Vannan *et al.* 2025).

The input data files are the list of individual transcripts with spatial locations in each tissue sample. Starting with the list of spatially detected transcripts, the following general major steps are involved in the process: (i) constructing spatial RNA graphs based on a selected radius threshold for each sample, with optional tissue tiling; (ii) sampling subgraphs from the RNA graphs for each sample or tile; (iii) merging subgraphs across samples and training the selected GNN model using the merged graph; and (iv) performing inference to get latent embedding for transcripts in individual samples. A variation of steps 2 and 3 is to skip subgraph sampling and merging, instead creating a batched loader of tile graphs that are directly used for training (as demonstrated in Case Study 2). In the unified embedding space, transcript-based clusters (or molecular niches) can be detected through a selected clustering algorithm. Utilizing the PyG framework, we implemented the SpatialRNA package that provides convenient construction and processing of graphs, facilitating large-scale iST data analysis.

### 2.1 Build sample graphs

Spatial RNA graphs are constructed using a radius-based approach for each sample using individual RNA transcripts as nodes. Edges are added between nodes with distances smaller than the selected radius threshold. For large tissue samples when the entire tissue graph does not fit in memory, tiles can be created along the chosen axis, such as along the *x* or *y* axis. The number of tiles is set by the user and each tile is also padded with extra margins to include the required spatial neighbours of transcripts located on the edge of the tile boundaries (to avoid artificial edge effects). A binary label is added to record which transcripts are from the core area or the padded area. Initialisation of the input feature vector for each node/transcript is set as the integer gene ID, which helps to reduce storage costs of the constructed graph. In the training and inference stages, integer IDs are mapped to one-hot-encoding vectors per batch of data.

### 2.2 Build sample subgraphs

Subgraphs are sampled from each sample graph using ‘LinkNeighborLoader’ from the PyG library in either a node-link-based or link-based manner. The node-link-based approach, first adopted in the Node2Vec model (Grover and Leskovec 2016) samples a fixed number of seed nodes first, and a fixed number of random walks are created from seed nodes to reach the first layer neighbours. Nodes reached by these walks are included in the subgraph along with their neighbours that are within a certain number of ‘hops’. Here, ‘hop’ refers to the distance to a neighbour measured by the number of steps required to reach the neighbour. Alternatively, link-based subgraph sampling randomly samples a chosen number of edges, and nodes reached by these edges along with their neighbours within a certain number of hops are included. We compared the two subgraph generation approaches and found them to not differ significantly under different GNN architectures (see Supplementary Material, available as supplementary data at *Bioinformatics* online).

As processing each tile or sample is independent from another, tile- or sample-based processes can be run in parallel (i.e. in multiple separate processing jobs), which includes the sample graph and subgraph construction, and embedding inference.

### 2.3 Model training

PyG library offers predefined ready-to-use GNN layers and GNN models (with in-built GPU acceleration supported through the PyTorch API) such as GraphSAGE (Hamilton *et al.* 2017), Graph Attention Networks (GAT) (Veličković *et al.* 2018), and GATv2 (Brody *et al.* 2022), making it convenient to experiment with different architectures as well as construct customized GNNs. Once the GNN model is configured, the model is trained using the merged subgraphs (Case study 1) or batched-loaded tile graphs (Case study 2). Mini-batching from the loaded graph is performed for model training via edge label prediction.

### 2.4 Inference on embeddings of individual transcripts

Inference on embeddings is performed for all nodes in the tile graph (in a mini-batch manner), but only nodes in the core area of each tile are included in the final output embedding matrix. The embedding matrices from multiple tiles originating from one tissue sample can be stacked to derive the embedding matrix for the full list of transcripts in the sample.

## 3 Case studies

To demonstrate the usage of SpatialRNA we conducted two case studies analysing spatially detected molecules from both lung tissues and a ovarian cancer tissue generated from the Xenium platform (10x Genomics). Full set of workflow and required scripts along with documentation can be found at https://ruqianl.github.io/spatialrna_docs/. The computational resources for both studies were recorded and handled efficiently on a common HPC system (Figs 1 and 2, available as supplementary data at *Bioinformatics* online).

### 3.1 Case study 1: Human pulmonary fibrosis dataset

We obtained the list of detected transcripts from 343 genes in 45 lung tissues processed with 10x Xenium from GEO with accession number GSE250346 (Vannan *et al.* 2025). We then processed each sample individually (without tiling) and created the sample graph using a radius threshold of 3.0 (microns). We subsequently sampled subgraphs from individual samples in a node-based fashion, which sampled 5000 seed nodes and performed 5 random walks per seed node for positive edge sampling. Nodes in the set of sampled edges along with 2-hop neighbours were kept in the subgraph. Subgraphs were then joined for GNN training. We constructed a 2-layer GATv2 (Brody *et al.* 2022) model as implemented in torch.geometrics (v2.6.1), and trained the model using the joined subgraphs. Using the trained GNN model, we predicted the embeddings of nodes (individual transcripts) across 45 tissue samples. Lastly, we jointly clustered transcripts across all samples using their embeddings with a Gaussian Mixture model. To demonstrate that spatial niches capture multicellular structure, we obtained the original cell type labels (Vannan *et al.* 2025) and calculated the cell type proportion assigned into each identified molecular niche (Fig. 1d and Fig. 3, available as supplementary data at *Bioinformatics* online). Importantly, these niches both capitulate the known biology of the lung [e.g. separately segmenting airways (T3, T9) versus alveolar structures (T6, T8)] (Fig. 1d, ij) and provide additional insights relevant to disease [e.g. a disease-enriched macrophage immune niche (T5)]. While SpatialRNA can identify and segment multicellular niches, it also provides opportunities for segmentation-free analysis of cell types via cell type decomposition using a reference dataset (Cable *et al.* 2022), or completely cell-free interpretation of results by applying gene proportion testing to compare niches (Figs 4–7, available as supplementary data at *Bioinformatics* online).

### 3.2 Case study 2: Human ovarian cancer sample with 5K gene panel

As a demonstration on state-of-art large-scale iST data, we analysed the human ovarian cancer sample processed on Xenium with a panel of 5000 genes from 10× Genomics using our method. Using SpatialRNA, we first tiled the tissue to 100 tiles along the *y* axis. We created RNA spatial graphs for individual tilesusing radius threshold as 3 microns. We then trained a 2-layer GraphSAGE model over the graph data of the 100 tiles. To fit the large-scale data into CPU memory, we used ‘SpatialRNAOnDiskDataset’ to help with iterating and batched loading of tile graphs (batch size 3). We then obtained molecular niches the same way as for the lung samples and compared the molecule niches with the cell group labels obtained from the reference source (Figs 8–11, available as supplementary data at *Bioinformatics* online) revealing that the molecular niches recover regions of heterogenous tumour and non-tumour tissues.

## 4 Comparing workflow variations and other methods on simulation data

The other leading segmentation-free tool for analysing iST data most comparable to SpatialRNA is FICTURE (Si *et al.* 2024). Like SpatialRNA, FICTURE is also highly scalable, but it differs in output (pixel-level factor assignments that are not easily aggregated to cell-level) and unlike SpatialRNA does not natively offer multi-sample integration or tiling for large datasets (Table 1, available as supplementary data at *Bioinformatics* online). While both tools identify multicellular niches, a realistic multicellular spatial data simulation is challenging. To compare them directly and efficiently, we tested the performance of the SpatialRNA workflow on a simple one-sample dataset of only 10 cell types simulated using FICTURE’s simulation method and tested recovery of these cell types as individual niches/factors (Supplementary Material, available as supplementary data at *Bioinformatics* online). We found that the SpatialRNA-workflow recovered true cell types robustly with different numbers of sampled edges across radius 1 and 3 [adjusted rand index (ARI) score > 0.9; Fig. 12, available as supplementary data at *Bioinformatics* online], which was on par with FICTURE (Si *et al.* 2024). To evaluate the performance of different configurations within the SpatialRNA-GNN workflows, we compared multiple workflow variations, including different GNN architectures (e.g. GAT, GATv2) trained on merged subgraphs sampled by the node-link-based and link- based approaches (Figs 13 and 14, available as supplementary data at *Bioinformatics* online) or trained on the batch-loaded on-disk tile graphs (Fig. 15, available as supplementary data at *Bioinformatics* online). These evaluations were performed on a multi-sample simulation dataset comprising 10 tissues and 24 cell types (Supplementary Material, available as supplementary data at *Bioinformatics* online). We found the subgraph sampling approaches did not differ significantly, and the GNN pipeline with GraphSAGE model achieved the best performance when training on batch-loaded tile graphs (Fig. 16, available as supplementary data at *Bioinformatics* online), which outperformed the FICTURE (Si *et al.* 2024) and GraphST (Long *et al.* 2023) by ARI scores.

## 5 Conclusion

We presented the open-source Python package SpatialRNA for convenient and efficient processing of spatially detected transcripts from iST data, which allows seamless integration with GNN methods in the PyG framework, making molecule-based analysis ready for ongoing GNN advancement. We demonstrate the usage of the package and the analysis workflow through two case studies that analysed iST datasets and demonstrated scalability on datasets with large numbers of samples (Case Study 1) or high sample areas and an expanded gene panel (Case Study 2). We showed that the molecule-based niches are biologically meaningful when correlating with cell-based results or using reference-cell-type-based decomposition, which also provide biological interpretation for the spatial niches that can be fed into downstream analyses (see Vannan *et al.* 2025). When there is no cell-based analysis, the overrepresented genes in each niche can be identified and used for interpreting the identified niches along with spatial organization. While both case studies were conducted on datasets from the Xenium platform, the method generalizes to other datasets with a similar format, that is features (e.g. mRNA or protein) with spatial locations. Additionally, the radius-based graph construction can be readily extended to 3D space. The package is currently designed to focus on molecule-based inputs, but can be applied to cell-based inputs with adaptation. Lastly, it is expected to tune parameter configurations for specific datasets, aligning with each specific analysis goal. For example, the radius thresholds used for constructing graphs control the size of spatial neighbourhoods for spatial smoothing, and increasing radius increases the level of spatial smoothing. The size of the GNN model (e.g. hidden layer sizes) and the size of sample neighbours are also expected to increase with increasing complexity in the dataset.

## Supplementary Material

btaf659_Supplementary_Data
