# Supplementary material for: SpatialRNA: a Python package for easy application of Graph Neural Network models on single-molecule spatial transcriptomics dataset
Source: Bioinformatics. 2025 Dec 13;42(1):btaf659. doi: 10.1093/bioinformatics/btaf659 (PMC12777972; doi:10.1093/bioinformatics/btaf659)
Supplement: btaf659_Supplementary_Data [file btaf659_supplementary_data.zip › SpatialRNA_supplementary_material.pdf]

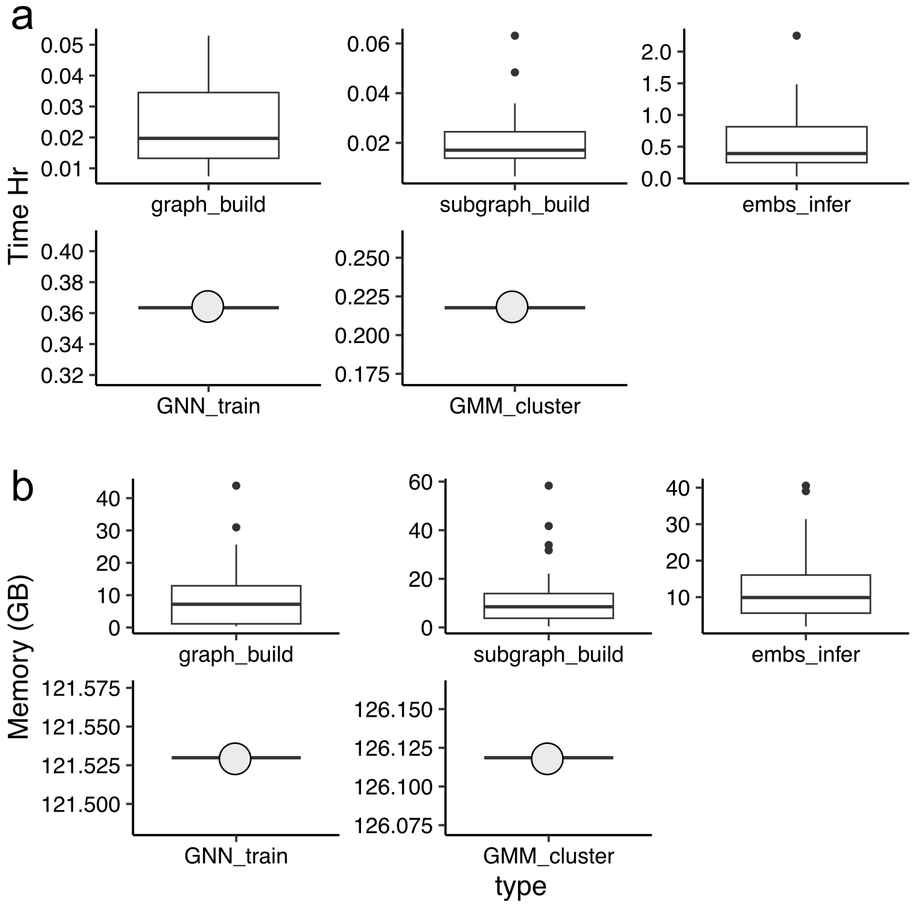

Fig. S1: a) Runtime and b) memory usage measurements for each processing step involved in the GNN-SpatialRNA workflow on 45 lung tissues. Processing steps include 1) graph_build: create spatial RNA graph per lung sample with radius as 3 microns. 2) subgraph_build: sample a subgraph per tissue graph. Samples 5,000 root nodes per tissue graph, each with 5 edges (totalling 25,000 edges). Nodes include their 2-hop neighbours, with all first-hop neighbours retained and a maximum of 50 sampled second-hop neighbours per node. 3) GNN_train: train the 2-layer GATv2 model with a total of 5 epochs over the joined graph from tissue subgraphs. 4) embs_infer, obtain transcript embeddings per lung sample. 5) GMM_cluster, apply GaussianMixtureModel clustering on all transcript embeddings from 45 tissues, deriving 12 transcript clusters. Boxplot shows the resources consumed for 45 individual samples. Benchmarking was performed on a high-performance computing cluster running Rocky Linux 9.6 (Linux kernel 5.14). CPU-only jobs were executed across nodes with AMD EPYC 9534 (64-core), AMD EPYC 7402 (24-core), or AMD EPYC 7543 (32-core) processors. GPU jobs, including GNN model training and PyCave-based clustering, were performed on GPU nodes equipped with NVIDIA Quadro RTX 8000 GPU cards (48 GB GPU memory). Each GPU job used a single GPU.

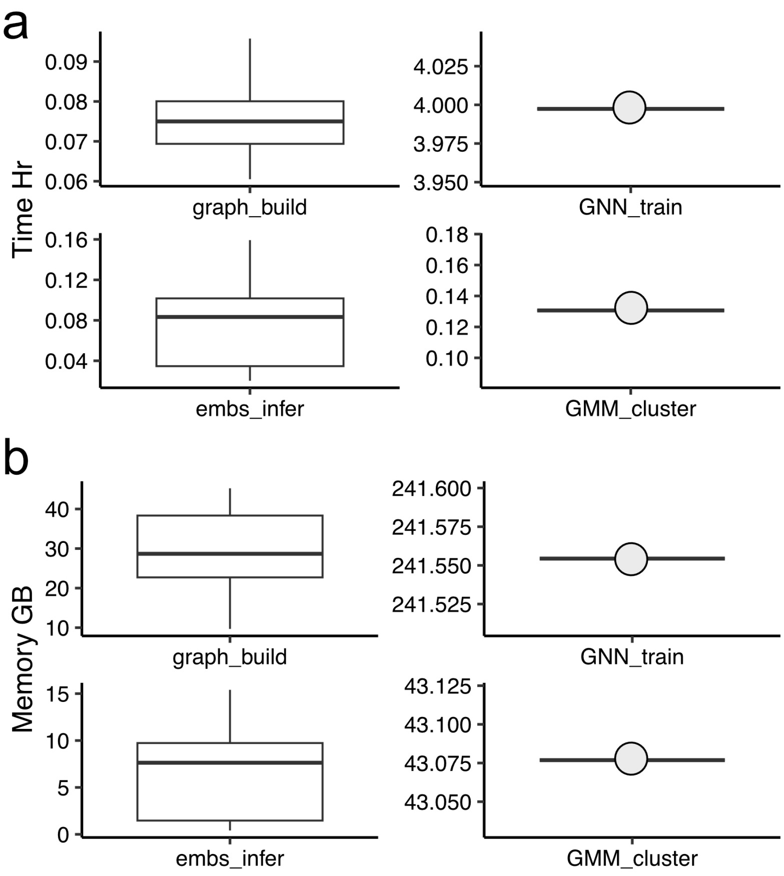

Fig. S2: a) Runtime and b) memory usage measurements for each processing step involved in the GNN-SpatialRNA workflow on the Xenium 5K ovarian cancer tissue. Processing steps include 1) graph_build: Tile tissue along Y axis and generated 100 tiles; Create spatial RNA graph per tile with radius as 3 microns. 2) GNN_train: train the 2-layer GraphSAGE model with a total of 10 epochs over the batch-loaded tile graphs (batch size 3). 3) embs_infer, obtain transcript embeddings per tissue tile. 4) GMM_cluster, apply GaussianMixtureModel clustering on all transcript embeddings from the whole tissue deriving 15 clusters. Boxplot shows the resources consumed for 100 individual tiles. System specifications were the same as in Fig. S1

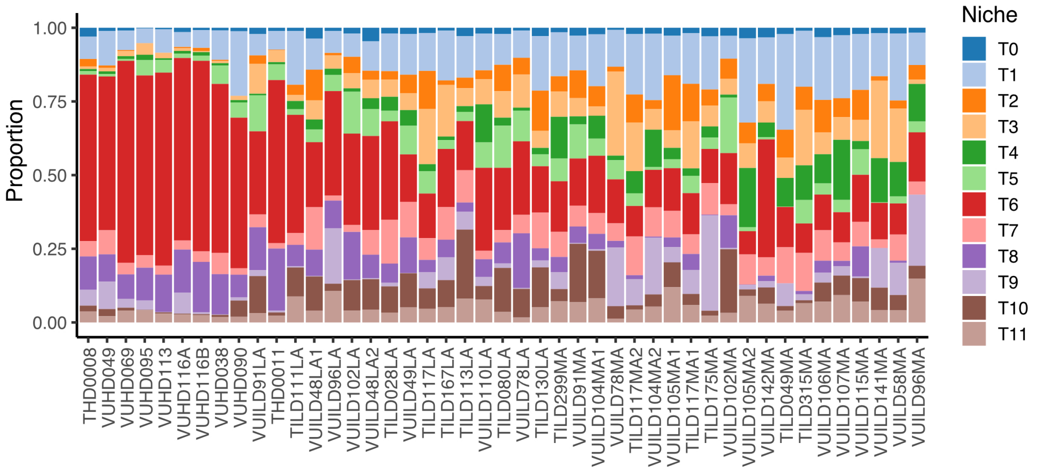

Fig. S3: Proportion of transcripts in each niche across 45 lung tissues. From left to right, the samples were ordered by their percentage of pathology (scored disease severity) from healthy samples to most severe fibrotic samples. See also Fig. 1d

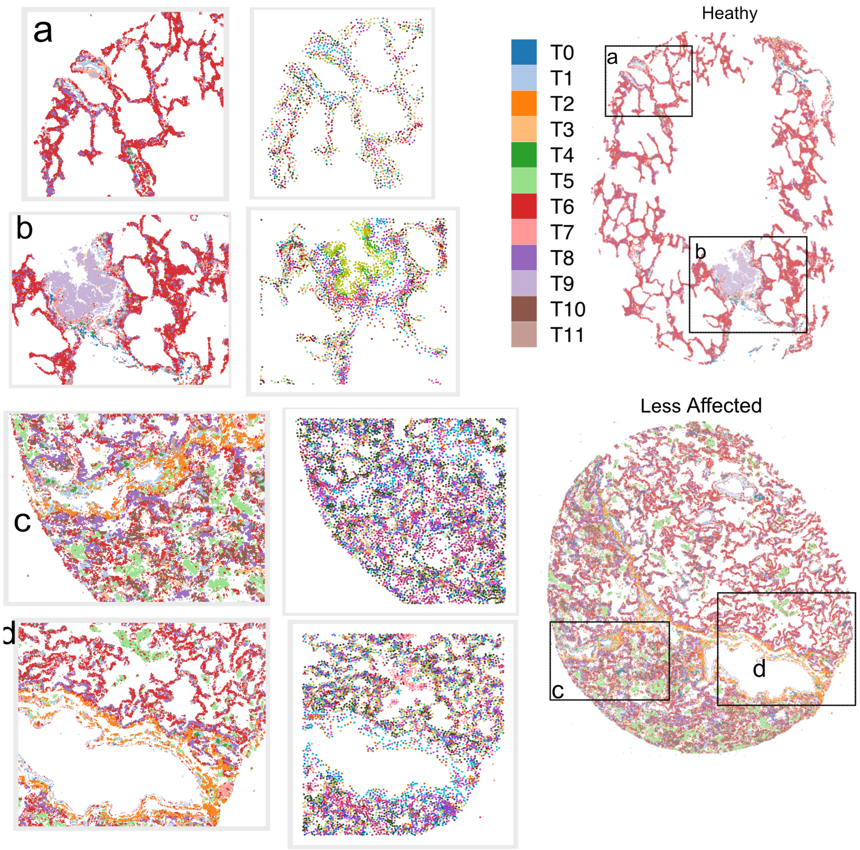

Fig. S4: Hex bin visualisation of molecular niches and cell types in 2 lung tissues, a healthy tissue on the top and a less fibrotic tissue on the bottom. Two zoom ins on molecular niches and cell types were shown for each tissue. a,b from a healthy tissue. c,d from an affected tissue with the top right hand less affected than the bottom half. Overall, we see the healthy tissue was mostly characterised by Niche T6 - interconnected cell types with homogenous spatial structure in healthy lungs, whereas the affected fibrotic sample showed more heterogeneous structures. a) Shows a lumen-like structure captured by Niche T6 and T1 (inner light blue layer of cells). b) Shows the airway epithelium captured by Niche T9. c) demonstrates a remodelled fibrotic lung with drastically changed organisation of cell types. d) Shows the fibroblast layer captured by Niche T2. c,d also showed the accumulation of macrophages in the alveolar spaces captured by Niche T5.

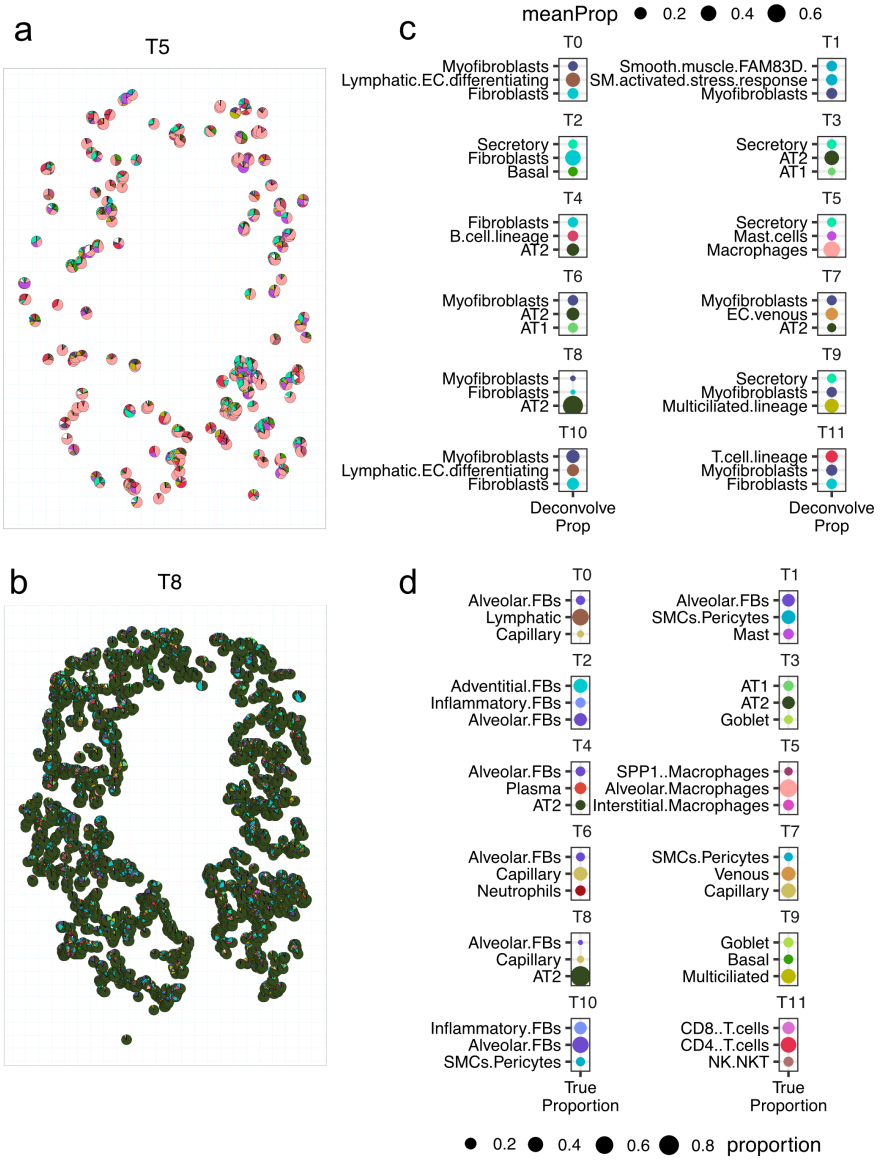

Fig. S5: RCTD analysis on one healthy lung sample from case study 1. a),b) Deconvolved cell type proportions in tiles (10 by 10 micron) of niche T5 and T8 with pie chart visualisation by RCTD. c) Top 3 deconvolved major cell types in each niche. d) The true cell type proportions in each molecular niche obtained from the cell-based analysis

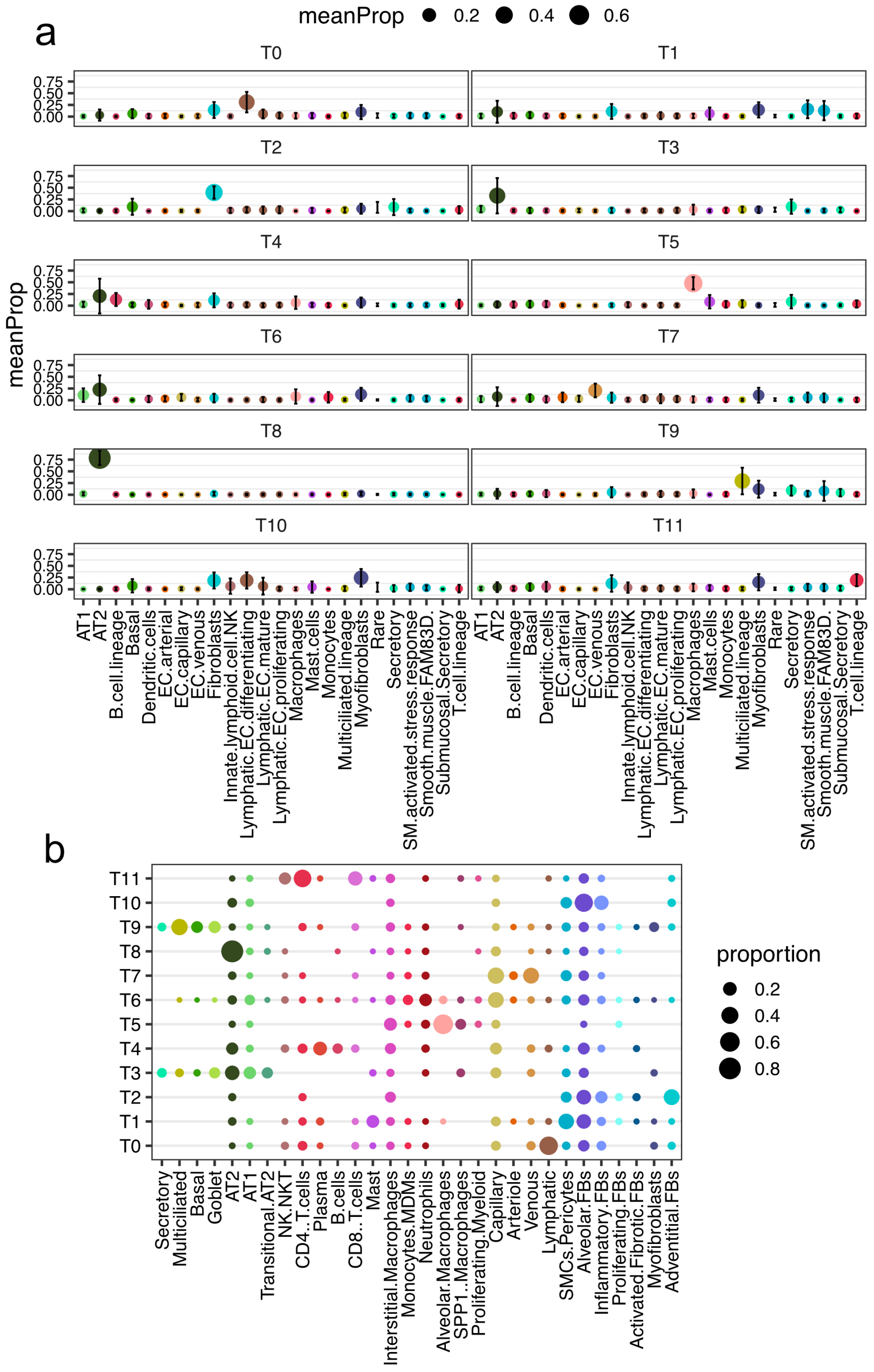

Fig. S6: Full results of RCTD analysis on one healthy lung sample from case study 1. a) Deconvolved cell type proportions in tiles (10 by 10 micron) in each niche. b The true cell type proportions in each molecular niche obtained from the cell-based analysis.

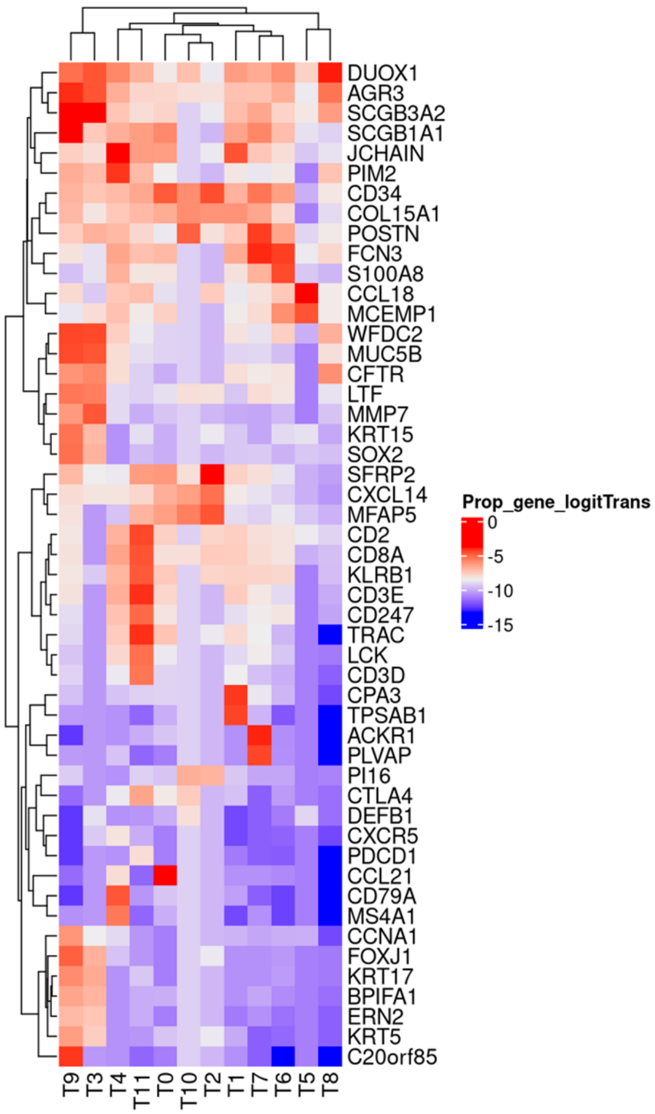

Fig. S7: Top 50 genes with highest variance among niches were selected and the gene proportions of them were visualised in the heatmap after logit transformation.

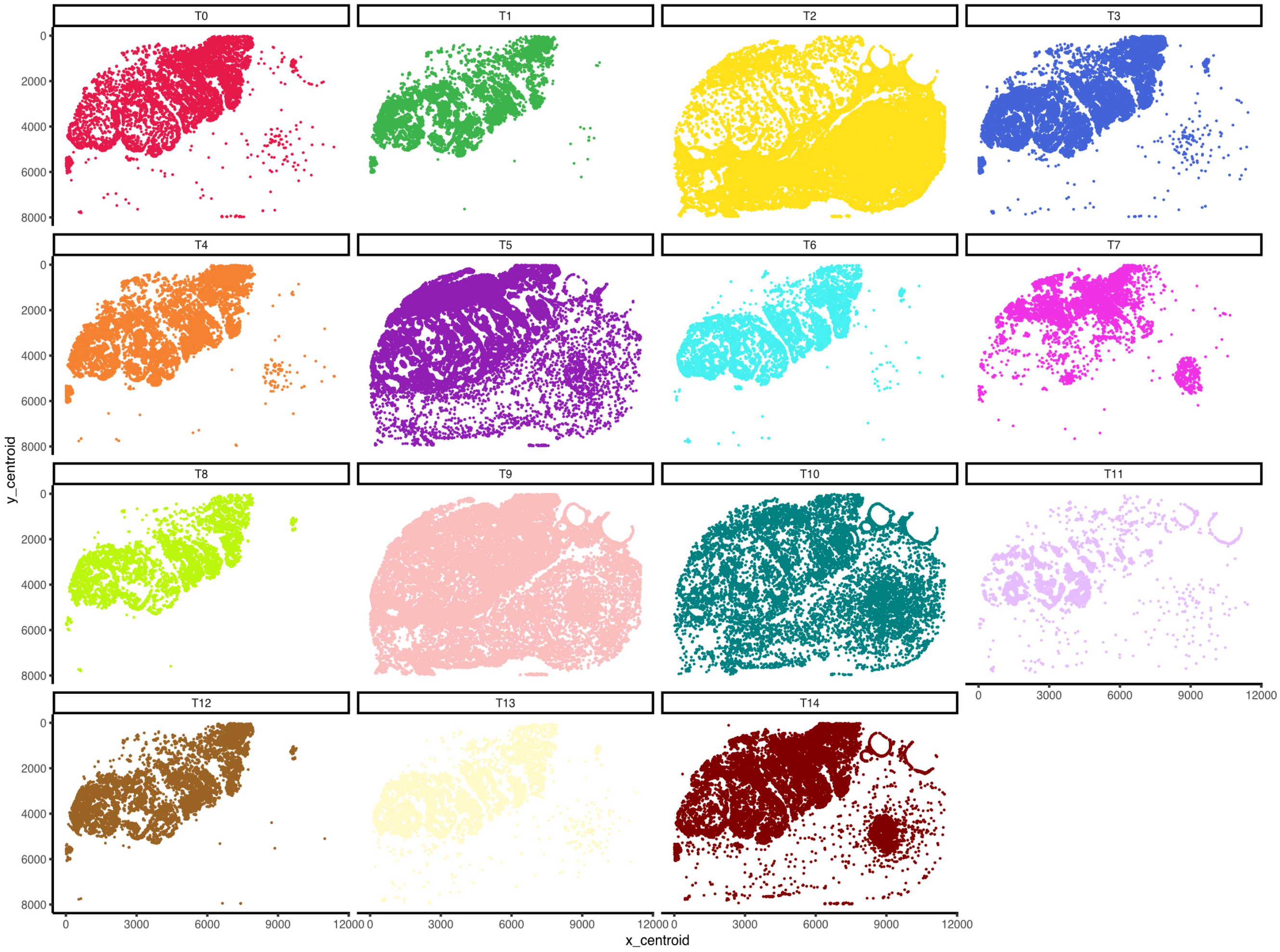

Fig. S8: The 15 identified molecular niches in an ovarian cancer tissue. The molecular niches were identified as demonstrated in case study 2, and the molecules were hex bin aggregated using a bin width of 5 (microns). Each hex bin is labelled with the major cluster labels among the spatially residing molecules in each hex bin. To compare with the cell annotations downloaded from 10x Genomics website, we obtained the cell centroids and assigned cell centroids to the hex bins by assigning cell centroids to their closest hex bins. We then transferred the hex bin label to cells.

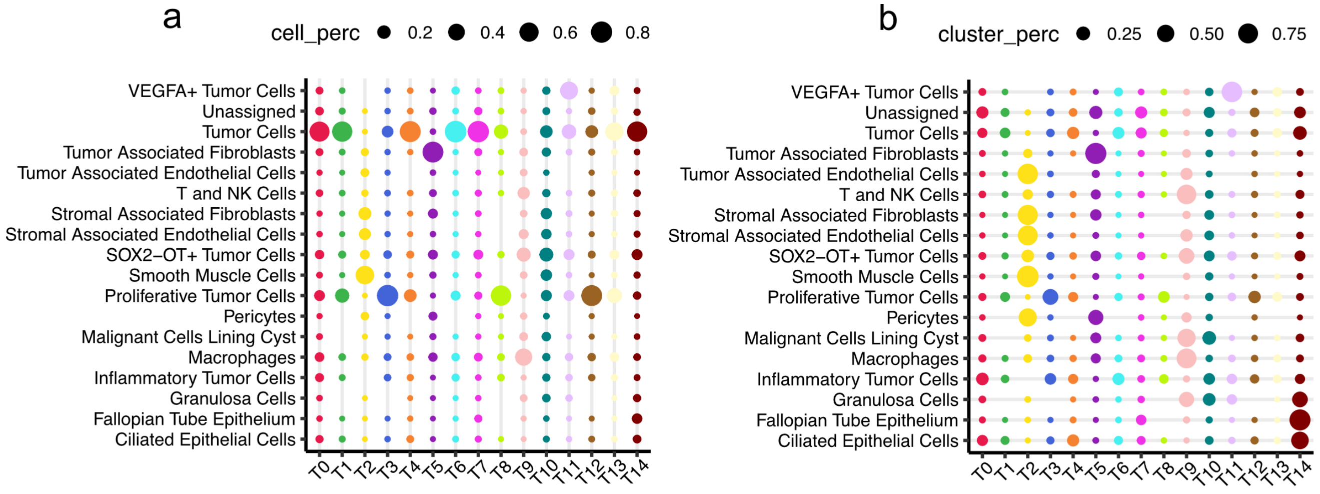

Fig. S9: Relations of cell annotation groups with molecular niches in the ovarian cancer tissue. Left panel shows the cell group composition for each molecule niche. Right panel shows the niche composition for each cell group. 'Tumor Cells' were captured by multiple niches such as T0, T1, T4, T6, T7, and T14. VEGFA+ Tumor Cells were captured in Niche T11. Proliferating Tumor cells in Niche T3, T8 and T12.

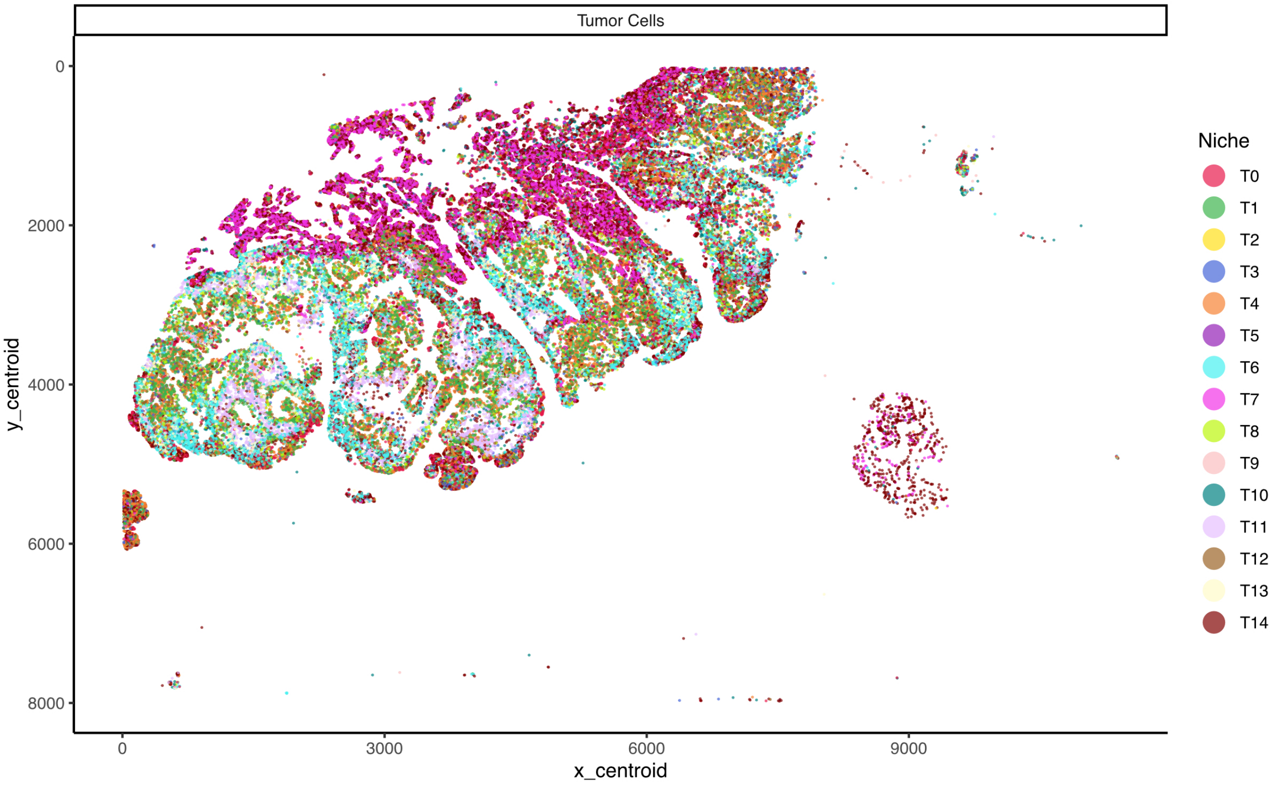

Fig. S10: Visualising molecular niches for 'Tumor Cells' only in the ovarian cancer tissue.

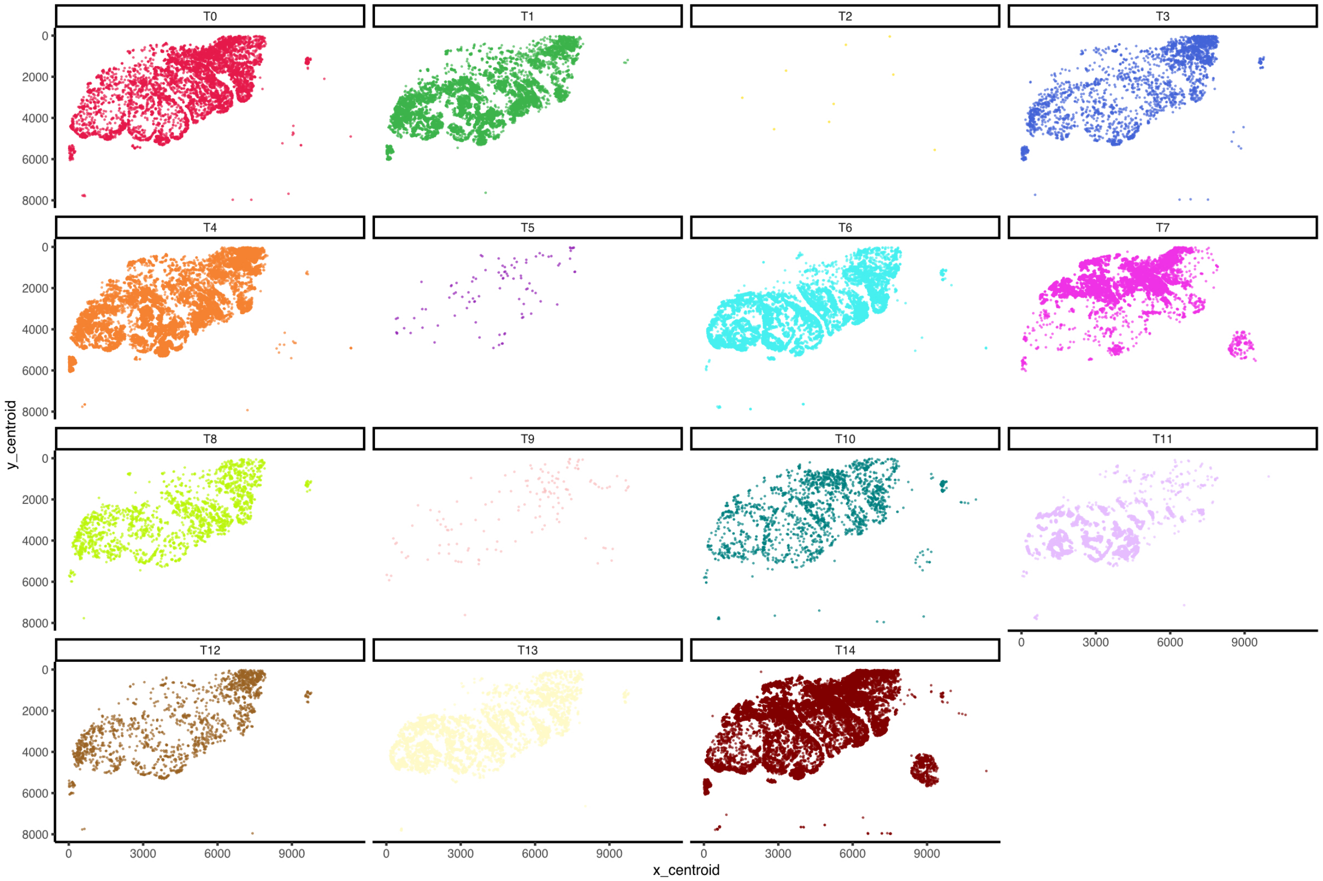

Fig. S11: Visualising 'Tumor Cells' only in the ovarian cancer tissue facet and coloured by their assigned molecular niche labels.

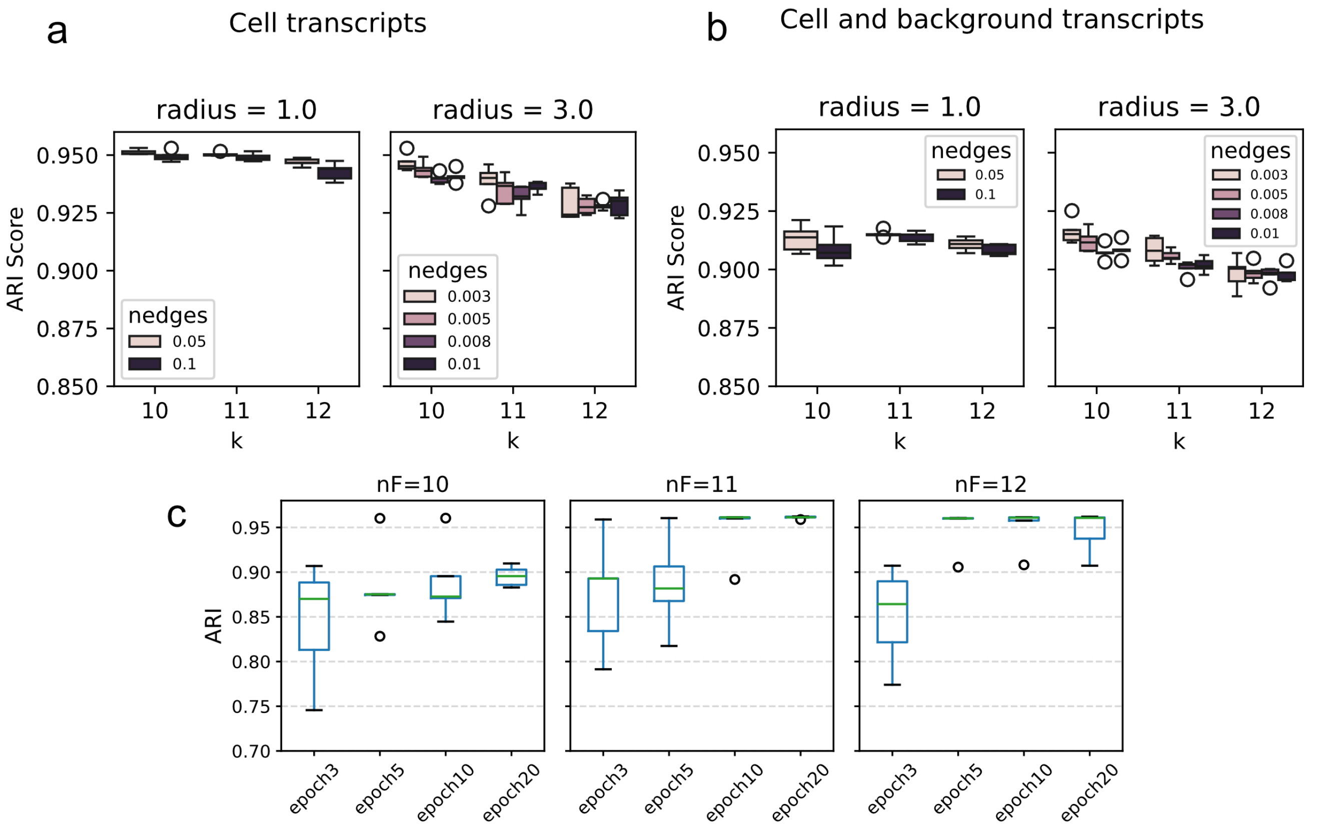

Fig. S12: ARI scores from SpatialRNA-GNN workflow and FICTURE on simulation dataset. Each boxplot shows the ARI scores from 5 repeated runs with different random seeds. a) ARI scores between cluster labels obtained by SpatialRNA-GNN workflow for K = 10, 11, 12. Column labels show the number of clusters and grouped by the number of sampled edges used for training. b) Same as a but for all transcripts i.e., transcripts included in simulated cells and in background. (total graph edges for radius 1 = 5,421,072, radius 3 = 45,941,972). For all GNN training runs, a 2-layer GraphSAGE model was used. A maximum of 20 epochs was applied with early stopping set to a patience of 3. Training terminates if the validation accuracy does not improve for 3 consecutive epochs. c) ARI scores between FICTURE results and simulated cell type labels for number of factors nF = 10, 11, and 12 after joining pixel-based results with input transcripts using post-processing tool spatua. 363,602 transcripts out of 557,521 total input transcripts were joined. Recommended parameters, identical to those in the FICTURE code repository, were applied

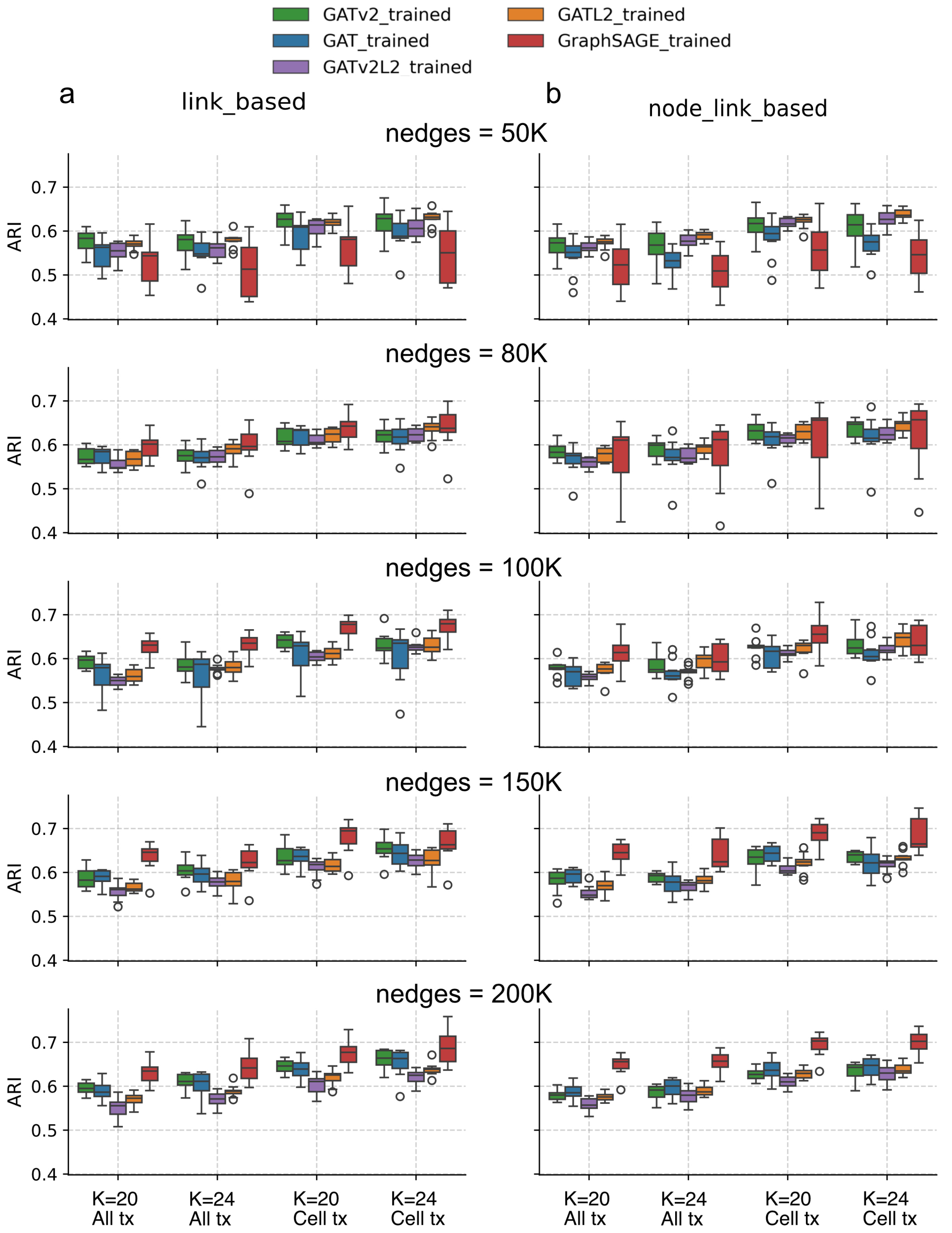

Fig. S13: ARI scores between the SpatialRNA-GNN workflow-derived cluster labels and the simulated ground-truth 24 cell type labels in 10 tissues. Each parameter setting was repeated 10 times with 10 different random seeds and plotted in each boxplot. a) The left column shows the results from link_based subgraph generation, and the right column shows the node_link_based subgraph generation. K=20 All_tx: overall ARI score for all transcripts clustered into 20 clusters.K=24 All_tx: overall ARI score for all transcripts clustered into 24 clusters. K=20 Cell_tx: overall ARI score for cell transcripts clustered into 20 clusters. K=24 Cell_tx: overall ARI score for cell transcripts clustered into 24 clusters. b) The same as a, but for subgraph inputs generated by the node_link_based method. nedges denote the number of sampled edges from each tissue that were kept to generate the subgraph for each tissue. GNN trainings were performed on the merged subgraphs from all tissues.

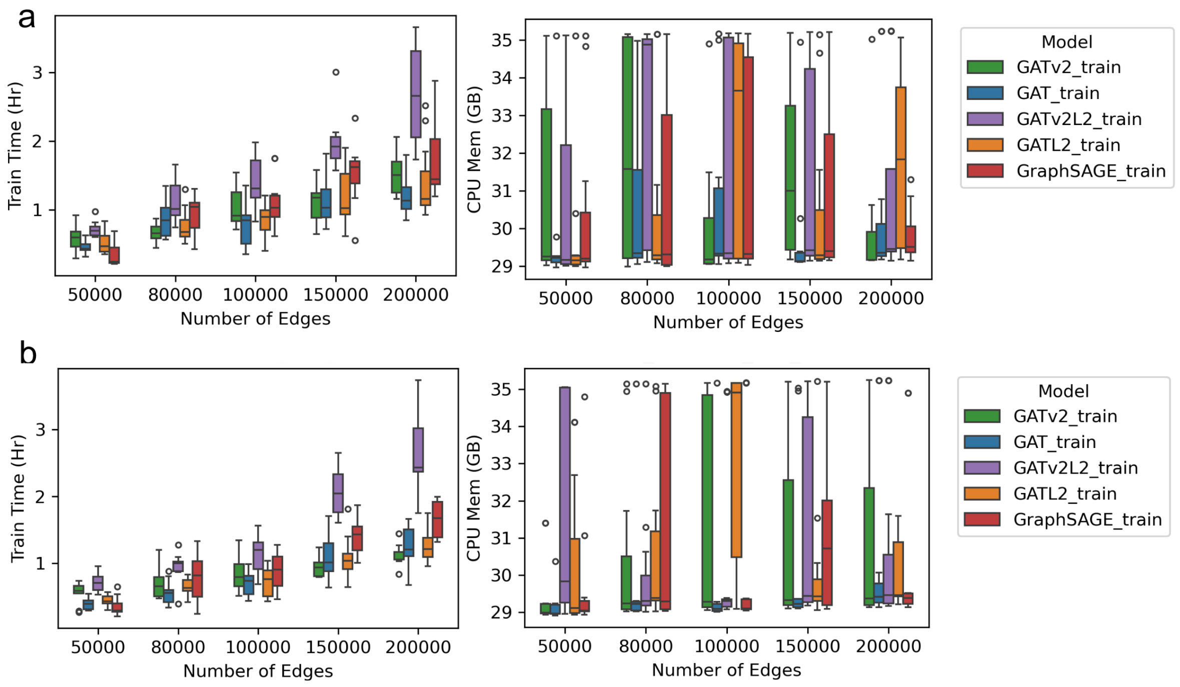

Fig. S14: Comparisons of computational resources, including training time and memory usages, consumed by the GNN runs using subgraph-based training presented in Fig. S13. a) link-based subgraph method b) node-link-based subgraph method

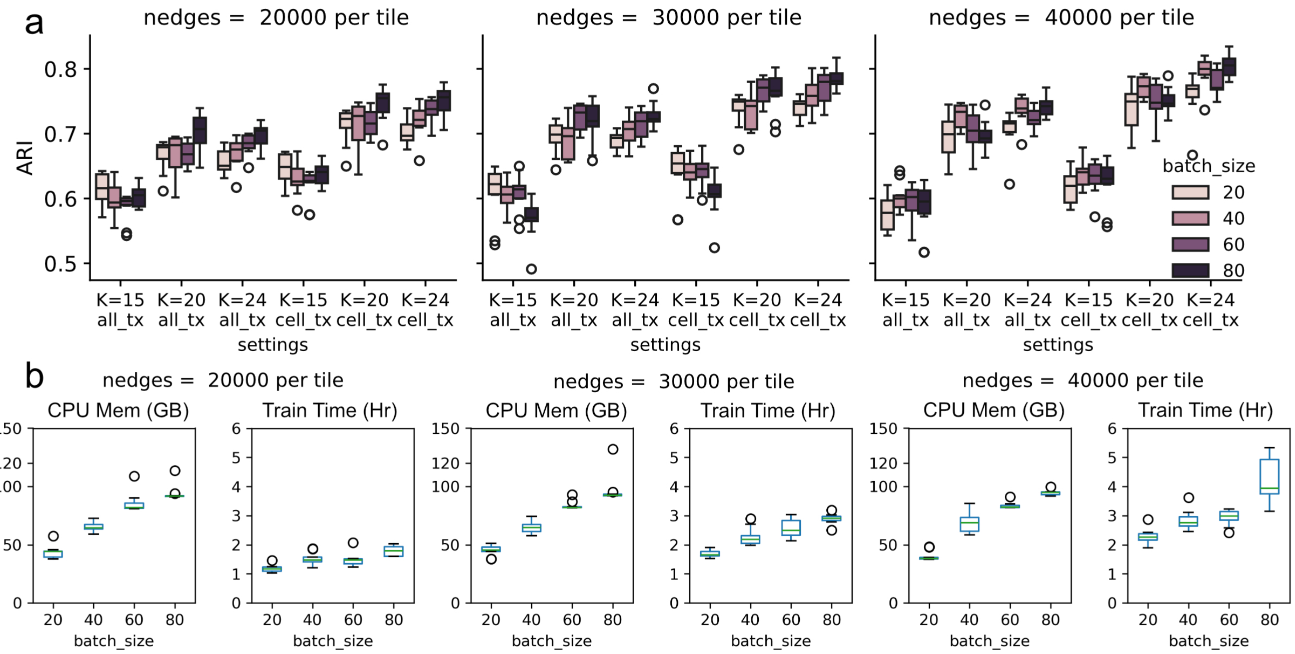

Fig. S15: Performance of training with batched-loaded tile graphs on the 10 simulated tissues. a) ARI scores from SpatialRNA-GraphSAGE workflow with training on the batched-loaded tile graphs. Ten tiles were created per tissue. Different batch sizes, and cluster number K were tested. all_tx, all transcripts in the tissues. cell_tx, transcripts included in the simulated cells. Each boxplot shows the ARI scores from 10 repeated runs with different random seeds. All models were trained for 10 epochs. b) The CPU RAM usages and training times for different batch sizes tested for nedges=20k, 30k and 40k per tile.

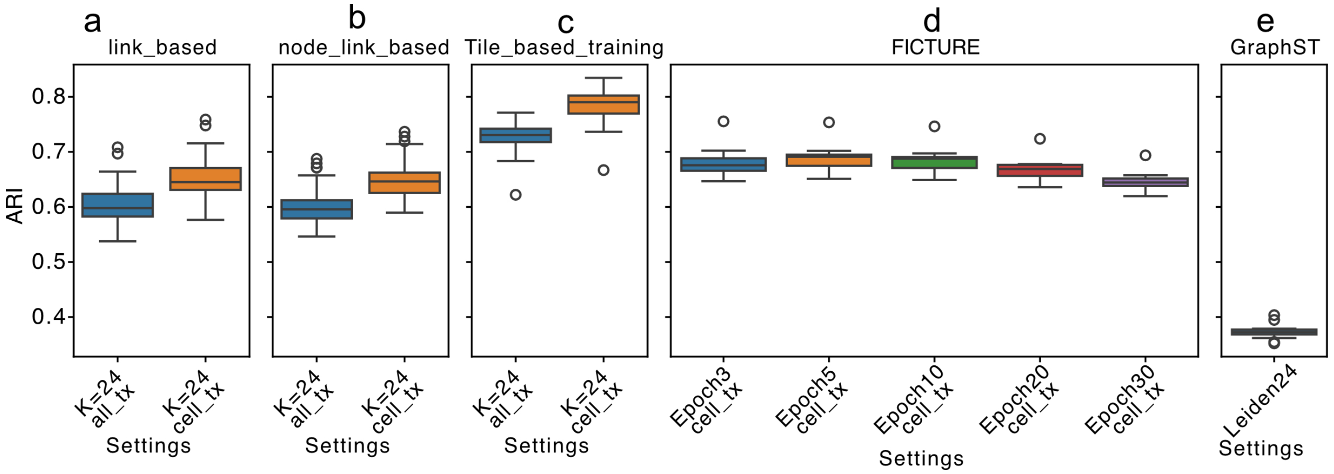

Fig. S16: ARI scores for variations of the SpatianRNA-GNN workflows, FICTURE and GraphST for detecting 24 different cell types on the 10 simulated tissues. a),b) The SpatialRNA-GNN workflow results using link-based or node-link-based subgraph generations with GraphSAGE model. The same subgraph generation settings from the nedges=200K result in Fig.S13. c) The SpatialRNA-GNN workflow with GraphSAGE model trained on batch-loaded tile graphs. The training settings were the same as the in Fig. S15a using number of training edges 40,000 per tile). all_tx: all simulated transcripts 5,575,210 across 10 tissues. cell_tx: transcripts included in cells 4,595,090. d) FICTURE's ARI results after joining pixel-level factor decoding results with the input transcripts using spatula (3,121,440 transcripts remained). Each epoch setting was repeated 10 times with different random seeds. e) ARI score between cluster labels derived from GraphST (10 repeats with different random seeds) and ground-truth cell type labels. GraphST takes cell-level expression and cell coordinates as input.

Cell type deconvolution on molecular niches using RCTD We applied RCTD [1] to transcripts in tiled molecular niches (10 by 10 tiles) in one healthy lung tissue (Fig. S5 and Fig. S6) to decompose reference cell types in the niches. The gene expression profile of the reference cell types was downloaded from the Human Lung Cell Atlas (HLCA core) project [2] and the annotation level 3 was used. We sampled 500 cells from each reference cell type label if the total number of cells available was more than 500. The raw count matrix of these reference cells was used to construct the reference cell type expression for running RCTD. For each molecular niche identified in the healthy lung tissue, we summarised the detected transcripts into gene expression profiles in 10 by 10 tiles across the tissue and estimated the reference cell type proportions across tiles using the RCTD method (Fig. S5a,b,c). Since we have the paired cell-level annotation [3], we can compare the RCTD-based cell type proportion estimation (Fig. S5c) to the true cell type proportion in the molecular niches (Fig. S5d). We see that the RCTD results agree with the true cell type proportion (Fig. S6). The top 3 major cell types by RCTD largely overlap with the true cell type proportion (Fig. S5).

Comparison with FICTURE on a simple simulation data for cell type detection We used the same simulation framework in FICTURE [4] and generated spatial transcripts across 500 um by 500 um area. The simulated data contains 10 cell labels arranged in 3 shapes with an average distance between cell centres 15 microns. Transcripts were simulated to a density of 4 transcripts per unit um^2. Background transcripts were also simulated with density as 20% of the transcript density in cells. Two cell types (Kuffer_cell and granulocytes) are scattered across the area while the remaining cell types are more restricted in local regions. We applied FICTURE on the simulated data using the suggested parameters provided in the code repository and varied the number of factors to 10, 11 and 12, as well as the number of epochs (Fig. S12)

FICTURE does not by default output transcript-level results and generates decoded grid-structured pixel-level results. However, FICTURE offers a post-processing tool *spatula* that joins transcripts to the pixels output from the main FICTURE method. We applied *spatula* to obtain transcript-level results after running the main FICTURE function. In this process, not all transcripts were able to be linked to pixels, likely due to the filtering step in the model (363,602 transcripts out of 557,521 total input transcripts were joined) (Fig. S12b) .

For the SpatialRNA-GNN workflow, we applied 2-layer GraphSAGE model, with radii 1 or 3. After obtaining the embeddings for all transcripts, we clustered the transcripts into 10, 11 and 12 clusters respectively (Fig. S12a).

As shown in Fig. S12 when deriving 10 clusters (the true number of cell types in the simulated data) using the two approaches, the SpatialRNA-GNN workflow outperformed FICTURE, achieving better and more stable ARI scores with overall ARI $>$ 0.91 compared to FICTURE's best score of 0.89. When using more factors than the number of cell types, FICTURE performed slightly better. Overall, on the simulation data, FICTURE had a slight advantage over the SpatialRNA-GNN workflow. Given that the simulation data were generated using the same framework as FICTURE, these results indicate that SpatialRNA-GNN still performs quite well.

Comparison of the subgraph generation methods and GNN models To assess which subgraph sampling methods are better for spatial transcripts data, we expanded the previous simulation data and generated 10 lung tissues, each with 10 different cell types and a total of 24 cell types across the 10 tissues. We obtained cell type expression profiles of these 24 cell types from a published study [5]. We applied the SpatialRNA-GNN workflow to the 10 tissues and identified 20 and 24 molecular niches, for which we calculated the ARI against the ground-truth cell type labels. We also varied the number of sampled edges per tissue for subgraph construction (denoted as nedges in Fig. S13 and Fig. S14). These sampled edges were used as positive edges for training, along with an equal number of negative edges sampled from the combined graph of all subgraphs. The sampled edges were split into 80% for training and 20% for validation. The same stopping criteria were applied for all runs, with a maximum of 20 epochs and early stopping with a patience of 3, which halts training if validation accuracy does not improve over three consecutive iterations. Overall, we found that the two subgraph sampling methods yield a similar performance. Using the simulation data, we were also able to evaluate the performance of different GNN models and the size of the subgraphs generated (i.e., nedges, Fig. S13), on which the model was trained on. Interestingly, we observed that four of the five tested models quickly saturated as subgraph size increased, whereas the fifth model, GraphSAGE, continued to benefit from larger subgraphs. This prompted us to investigate whether GraphSAGE's performance could be further improved when applied to the full graph.

Batched loaded tile graph for training Loading full graphs of tissue tiles, especially when integrating multiple samples, is highly memory-intensive on CPU RAM before mini-batching graphs on the GPU for model training. To address this, we implemented a new data loader in SpatialRNA that integrates well with the existing implementation framework and provides convenient access to on-disk tile graph for loading batched tile graphs stored on disk. We

then trained a 2-layer GraphSAGE model on batch-loaded tile graphs with varying batch sizes and calculated the ARI scores (Fig. S15a). We observed that indeed, with batched training on tile graphs, GraphSAGE achieved a higher ARI than using subgraphs. To validate that the batched tile graph loader controls the CPU RAM usage, we measure the memory usages for different batch sizes (Fig. S15b, Fig. S13, and Fig. S16) and confirmed small batch size consumes less memory. All training was run with 10 epochs.

Comparison with FICTURE and GraphST on the multi-sample simulation data Leveraging the multi-sample simulation data, we were also able to apply two other methods, GraphST and FICTURE, on the multi-sample simulation data for comparison and observed that the GraphSAGE result was better than both (Fig. S16) for cell type identification across multiple tissues. To apply FICTURE and GraphST on the 10 tissues jointly, we concatenated the transcripts from 10 tissues and added gaps of 15 microns, arranging them in 2 by 5 grids. We referred to the example script used in the simulation example in the code repositories of FICTURE, and ran the following steps,1) make_spatial_minibatch with batch size 500 and batch buffer 20, 2) make_dge to generate hexagon summary using width 12 with sliding step 2, 3) sorted hexagon by major axis X, 4) fit the model (with model_fit) with number of factor 24 and set the epoch to 3, 5, 10 and 20 respectively. 5) Transformed the fitted model to factor space for anchor hexagons with width 9 (with transform), 6) decode factor relevance for pixels (slda_decode) with precision 0.25. 7) Joining back the pixel-level results with input transcripts to assign transcripts with the top relevant factor using spatula. GraphST is cell-based method and requires cell segmentation, but we applied GraphST on this multi-sample data as well. For running GraphST, we summarised the gene expression per simulated cells and used the centroids of cells as spatial coordinates for GraphST and applied the leiden clustering method to derive 24 cell clusters. The 24 cell cluster labels were compared with the simulation labels for ARI score calculation. We repeatedly applied the process with 10 different random seeds.

References

[1] Cable DM, Murray E, Zou LS, Goeva A, Macosko EZ, Chen F, et al. Robust decomposition of cell type mixtures in spatial transcriptomics. Nature Biotechnology;40(4):517-26.

[2] Sikkema L, Ramírez-Suástegui C, Strobl DC, Gillett TE, Zappia L, Madissoon E, et al. An integrated cell atlas of the lung in health and disease. Nature Medicine;29(6):1563-77.

[3] Vannan A, Lyu R, Williams AL, Negretti NM, Mee ED, Hirsh J, et al. Spatial transcriptomics identifies molecular niche dysregulation associated with distal lung remodeling in pulmonary fibrosis. Nature genetics. 2025 Feb:1-12.

[4] Si Y, Lee C, Hwang Y, Yun JH, Cheng W, Cho CS, et al. FICTURE: scalable segmentation-free analysis of submicron-resolution spatial transcriptomics. Nature methods. 2024 Oct;21(10):1843-54.

[5] Natri HM, Del Azodi CB, Peter L, Taylor CJ, Chugh S, Kendle R, et al. Cell-type-specific and disease-associated expression quantitative trait loci in the human lung. Nat Genet. 2024 Apr;56(4):595-604.
